# 88. Letermovir as CMV Prophylaxis in Liver Transplant Recipients

**DOI:** 10.1093/ofid/ofaf695.034

**Published:** 2026-01-11

**Authors:** Emily A Siegrist, Phu Nguyen, Rita Wilson Dib, Joseph Sassine

**Affiliations:** OU Health, Oklahoma City, Oklahoma; OU Health, Oklahoma City, Oklahoma; University of Oklahoma, Oklahoma City, Oklahoma; University of Oklahoma Health Sciences Center, Oklahoma City, OK

## Abstract

**Background:**

Letermovir has emerged as a safe and effective antiviral agent for CMV prophylaxis in allogeneic HCT recipients and high-risk kidney transplant recipients. There is a paucity of data on the use of letermovir in liver transplant recipients. This study aims to compare letermovir to valganciclovir in terms of efficacy and safety for CMV prophylaxis in liver transplant recipients.

**Table 1:**
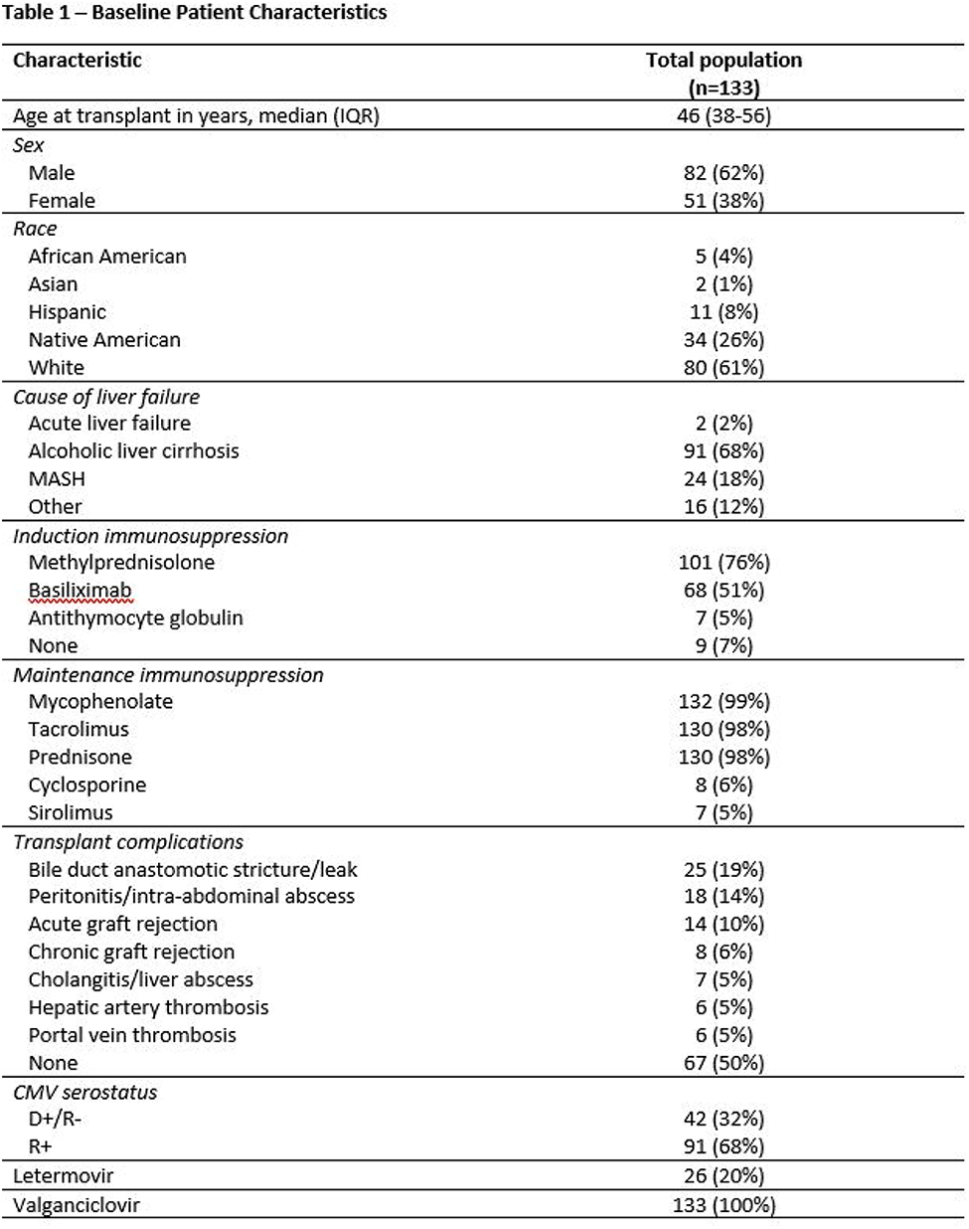
Baseline Patient Characteristics

**Table 2:**
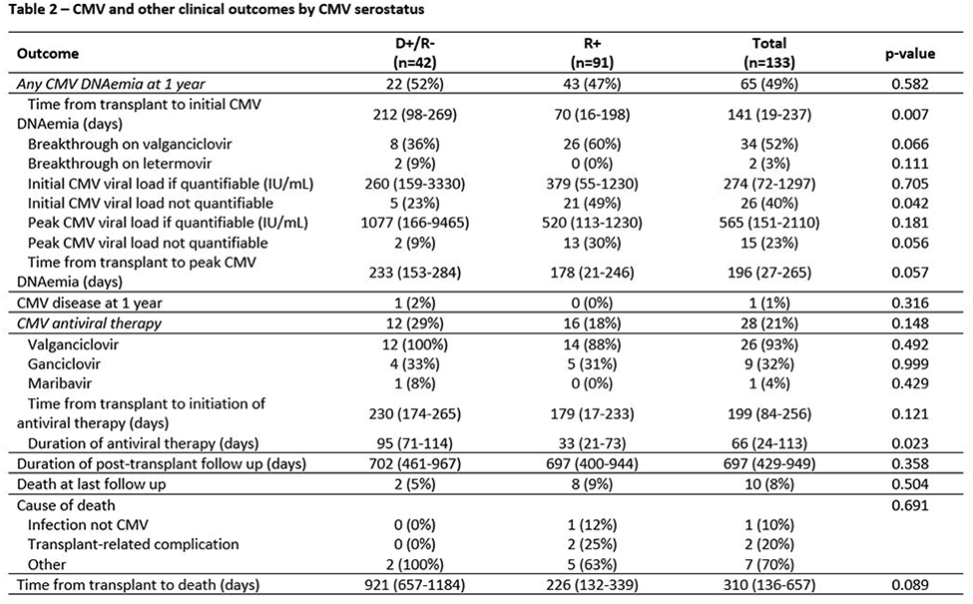
CMV and Other Clinical Outcomes by CMV Serostatus

**Methods:**

This single-center, retrospective cohort included all consecutive patients who received a liver transplant between January 1, 2021, and April 30, 2024. Patients who received more than one liver transplant were excluded. Statistical analysis was performed using SPSS.

**Table 3:**
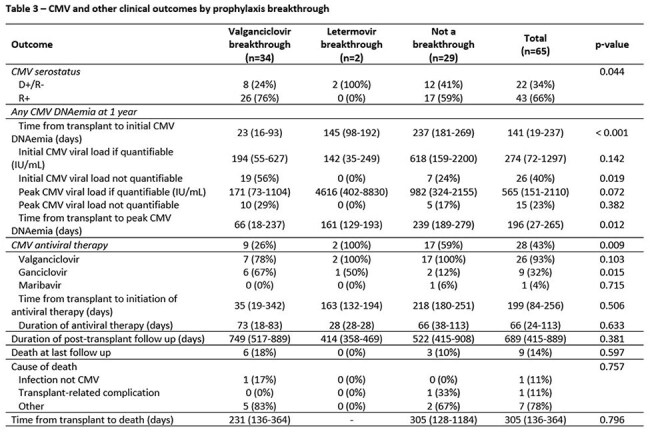
CMV and Other Clinical Outcomes by Prophylaxis Breakthrough

**Table 4:**
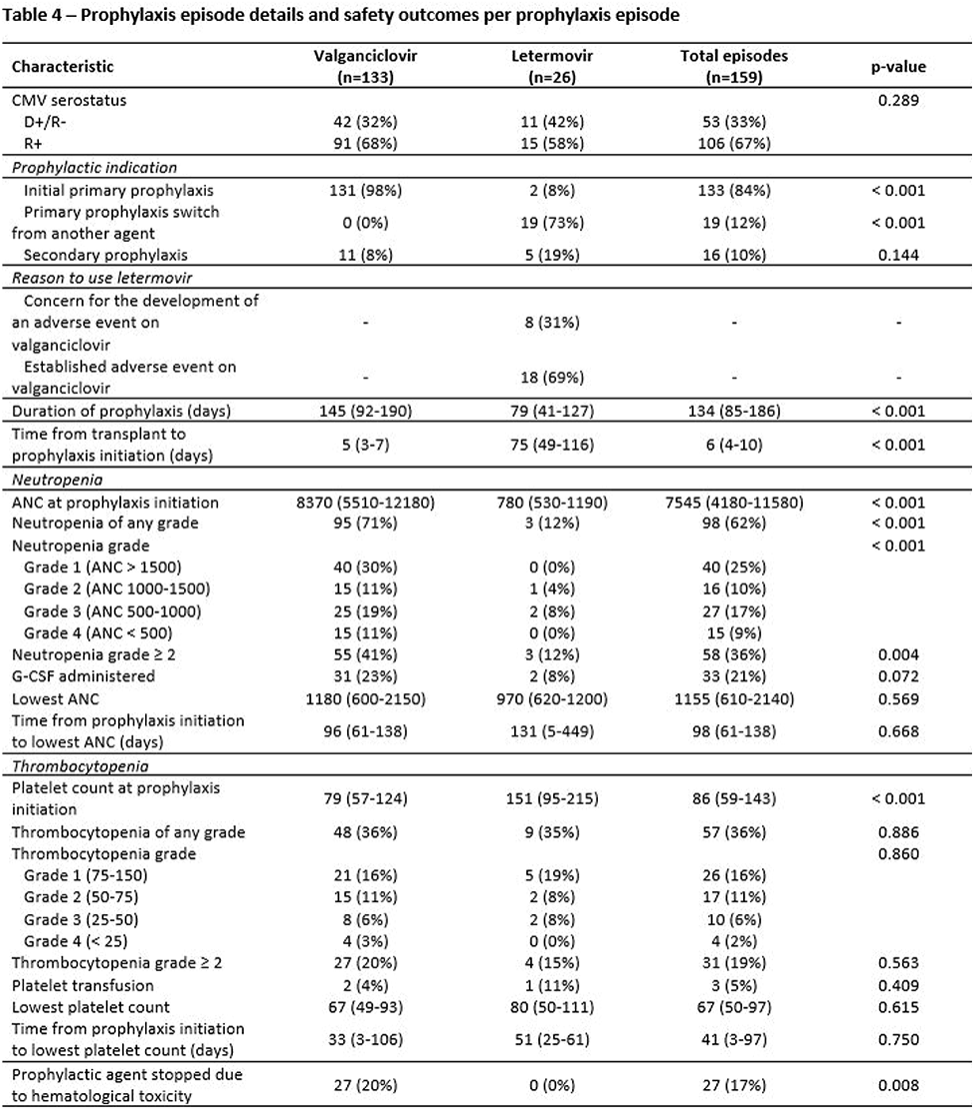
Prophylaxis Episode Details and Safety Outcomes per Prophylaxis Episode

**Results:**

A total of 133 patients met inclusion criteria, 42 (32%) were D+/R- and 91 (68%) were R+. All patients had received valganciclovir, and 26 (20%) patients received letermovir, most of whom were transitioned due to an established adverse event on valganciclovir. There were significantly fewer CMV DNAemia breakthroughs on letermovir (2, 8%) than on valganciclovir (34, 26%, p = 0.046) within the first year post-transplant. Only one patient developed CMV disease (colitis) and was on letermovir. The letermovir prophylaxis episodes had significantly fewer events of neutropenia of any grade (12% vs. 71%, p < 0.001), neutropenia of grade ≥ 2 (12% vs. 41%, p < 0.001), G-CSF use (8% vs. 23%, p = 0.072), and drug discontinuation due to hematological toxicity (0% vs. 20%, p = 0.008), compared to valganciclovir.

**Conclusion:**

Letermovir is a safer alternative to valganciclovir for CMV prophylaxis, and potentially more effective at suppressing CMV replication, in liver transplant recipients. Large prospective studies are needed to confirm these findings.

**Disclosures:**

All Authors: No reported disclosures

